# Diagnosis and Treatment of Plasmablastic Lymphoma in an Immunocompetent Patient: A Report of a Rare Case

**DOI:** 10.7759/cureus.87736

**Published:** 2025-07-11

**Authors:** Pyrus Bhellum, Rajat Goyal, Satyendra Khichar, Vikarn Vishwajeet, Harshita Yalla

**Affiliations:** 1 Department of General Medicine, All India Institute of Medical Sciences, Jodhpur, Jodhpur, IND; 2 Department of Pathology and Lab Medicine, All India Institute of Medical Sciences, Jodhpur, Jodhpur, IND; 3 Department of Diagnostic and Interventional Radiology, All India Institute of Medical Sciences, Jodhpur, Jodhpur, IND

**Keywords:** epoch, immunocompetent adult, plasmablastic lymphoma (pbl), plasmablastic myeloma, spine tuberculosis

## Abstract

Plasmablastic lymphoma (PBL) is a rare and aggressive subtype of diffuse large B-cell lymphoma (DLBCL), often associated with immunodeficiency states such as HIV infection. However, PBL can also occur in HIV-negative individuals, where it exhibits diverse anatomical involvement and poorer prognosis. This case presents a 50-year-old HIV negative male patient with progressive lower limb weakness and numbness due to a thoracic paravertebral lesion extending from T1 to T6, causing compressive myelopathy. Imaging raised concerns for malignancy, and histopathology confirmed PBL with positivity on IHC for CD38, CD138, and Cyclin D1. Given its aggressive nature, early intervention was critical. The patient underwent surgical decompression via T2-T6 laminectomy followed by chemotherapy with the EPOCH regimen (etoposide, prednisone, vincristine, cyclophosphamide, doxorubicin). After two cycles, significant symptomatic improvement was observed. HIV-negative PBL is associated with poor prognosis and high relapse rates, necessitating aggressive treatment and close monitoring. This case highlights the challenges in diagnosing and managing HIV-negative PBL, emphasizing the importance of multimodal treatment strategies, including surgical intervention, intensive chemotherapy, and follow-up.

## Introduction

Plasmablastic lymphoma (PBL) is an aggressive and rare form of DLBCL, which is associated with poor prognosis [1]. In 1997, Delecluse et al. described 16 cases of primary oral DLBCL with a special immunophenotype, of which 15 cases were positive for human immunodeficiency virus (HIV), and proposed the diagnosis of PBL for the first time [2].

Initially, PBL was predominantly considered to be associated with patients infected with HIV. However, over time, it has also been identified in various anatomical sites in HIV-negative individuals [3,4]. Unlike in HIV-positive PBL, which has a strong predilection for oral cavity involvement, the primary sites of HIV-negative PBL appear to be more heterogeneous.

In recent years, there has been a growing number of reported cases of HIV-negative PBL in several extra-oral locations, including the gastrointestinal tract, soft tissue, bone marrow, skin, lymph nodes, sinuses, lungs, and central nervous system (CNS) [5,6]. In 2016, PBL was classified by the WHO as an independent subtype of large B-cell lymphoma [7,8], which was associated with HIV and EB virus infections, or other immunodeficiency states, such as long-term use of immunosuppressants, solid organ transplantation, or age-related immune decline.

Extraoral spinal involvement with paravertebral extension and spinal cord compression is exceedingly rare and can mimic other spinal pathologies such as tuberculosis or metastatic disease, particularly in endemic regions. Given its aggressive course and poor prognosis, timely diagnosis and treatment are essential to improving outcomes.

This case highlights a rare presentation of HIV-negative PBL manifesting as thoracic compressive myelopathy in a middle-aged man with a background of partially treated pulmonary tuberculosis. Through this report, we aim to emphasize the need for heightened clinical suspicion, comprehensive diagnostic evaluation including histopathology and immunohistochemistry, and prompt initiation of systemic therapy in atypical spinal masses to avoid neurological deterioration and misdiagnosis.

## Case presentation

A 50-year-old man with a known history of pulmonary tuberculosis diagnosed six months back, for which he had taken anti-tubercular therapy (ATT) for three months and then discontinued, presented with complaints of bilateral lower limb numbness and weakness, along with difficulty in standing, which had been progressively worsening over the past month. The patient initially noticed left-sided lower limb numbness, followed by motor weakness, which later progressed to involve the right lower limb over a period of two months. This was associated with increasing difficulty in performing daily activities such as standing and wearing slippers. Two days before admission, he was unable to stand without assistance. There was no history of fever, trauma, seizures, upper limb weakness, difficulty in deglutition, voice changes, or bladder or bowel incontinence.

On examination, general physical findings were unremarkable, but on CNS evaluation, the tone was increased in both the lower limbs (right greater than left), and power was reduced bilaterally (2/5 in the right hip, 3/5 in the left hip, and 4/5 in the remaining joints of the lower limbs. Reflexes were exaggerated (3+ in the knees and ankles), and bilateral plantar reflexes were mute. Sensory examination revealed impaired proprioception localizing to the T7 level. Spinal tenderness was elicited at the T5 level, while other systemic examinations were normal. Routine investigations revealed anemia and raised CRP, while other blood investigations, including the liver function test, kidney function test, serum electrolytes, and serum calcium levels, were within normal limits. Viral markers were negative.

Based on clinical examination, a provisional diagnosis of insidious-onset, gradually progressive spastic paraparesis with upper motor neuron signs, including hypertonia, exaggerated reflexes, and bilateral lower limb weakness, along with a sensory level at T7 and spinal tenderness at T5, suggestive of thoracic compressive myelopathy, was made. Given the prior incomplete treatment for pulmonary tuberculosis and elevated inflammatory markers, a provisional diagnosis of Pott’s spine with secondary spinal cord compression was kept.

**Figure 1:**
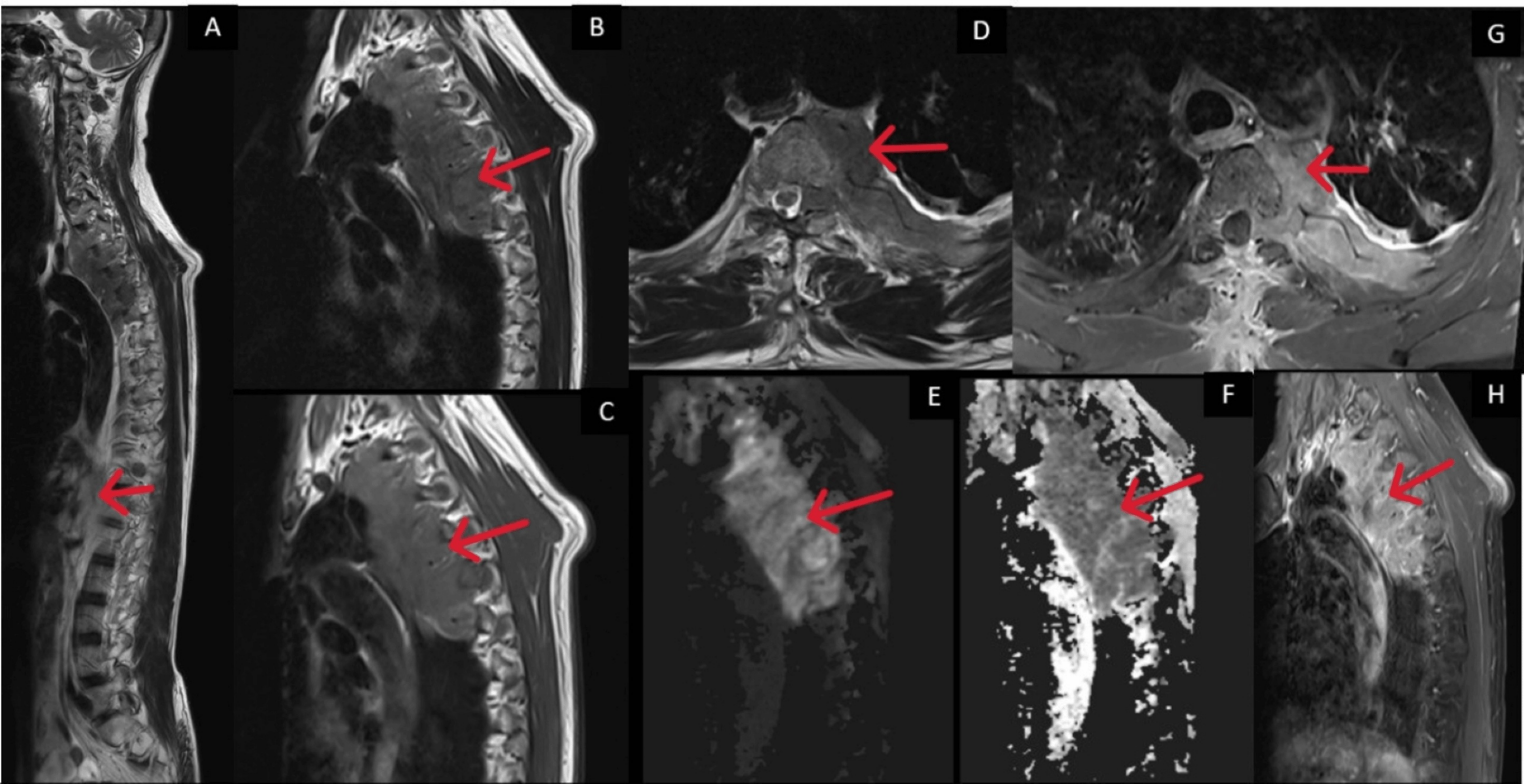
Sagittal and axial MRI images (T2W, T1W, DWI, ADC, and post-contrast; A to H) show a lobulated soft tissue lesion in the left upper thorax, located in the paravertebral and extrapleural region. The lesion shows restricted diffusion (E, F) and heterogeneous contrast enhancement (G, H). It extends medially through the left neural foramen into the epidural space, causing compression of the thoracic spinal cord.

**Figure 2:**
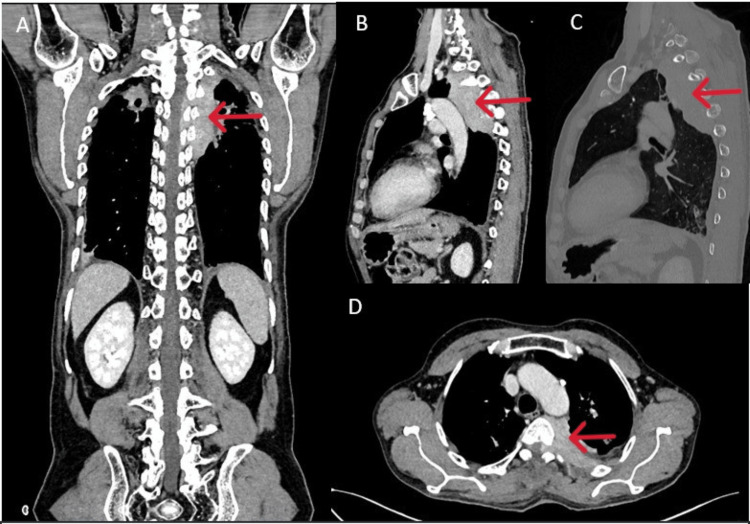
Multiplanar CECT images of the chest and abdomen (A to D) show an enhancing soft tissue mass in the left upper chest and in the paravertebral and extrapleural region from D1 to D6. The mass extends into the spinal canal through the neural foramina. There are no visible bone erosions in the nearby vertebrae or ribs (C), which suggests the infiltrative (permeative) pattern typical of lymphoma.

A CT scan of the spine was done, which revealed a hyperenhancing lesion in the left upper thorax, extending along the paravertebral region from T1 to T6 and into the left neural foramina (Figure 1). For better characterization, MRI spine was done, which revealed neoplastic or soft tissue thickening with intramedullary hyperintense signals at the T4 and T6 levels, suggestive of compressive myelopathy (Figure 2). This raised concerns for various differential diagnoses, including Ewing's sarcoma, hemangiopericytoma, and chondrosarcoma.

**Figure 3:**
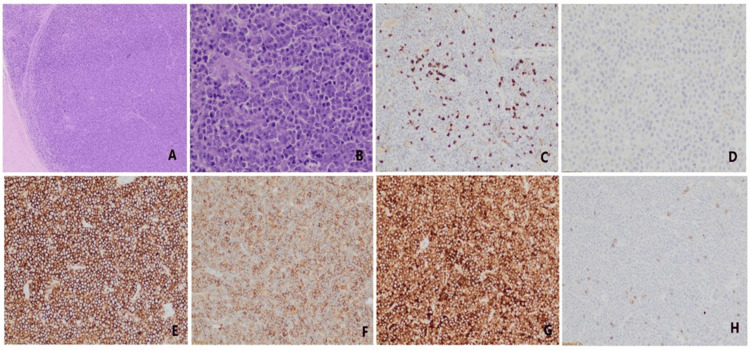
Histopathological images showing (A) Diffuse sheets of lymphoid cells with occasional fibrous septa running in between; (B) High magnification shows plasmablastic morphology of tumor cells with central prominent nucleoli and moderate amount of amphophilic cytoplasm; IHC of the lymphoid cells show absent expression of CD45 (C) and CD20 (D) while strong expression of CD38 (E) and EMA (F). Light chain restriction is observed in lymphoid cells where cells show strong expression of lambda (G) while kappa chain (H) is negative.

The patient underwent surgical decompression via T2-T6 laminectomy with tumor excision. Histopathology of the excised tissue showed diffuse sheets of atypical lymphoid cells with thin fibrous septae composed of large immunoblastic and plasmablastic cells with coarse chromatin and prominent nucleoli. A starry-sky pattern with brisk mitotic activity was noted. Immunohistochemistry (IHC) revealed positivity for CD38, CD138, EMA, Cyclin D1, and lambda light chain, with negativity for CD20, CD45, and CD56. CD5 highlights background T-cells. IHC and other histopathological features favored plasmablastic lymphoma over plasmablastic transformation of multiple myeloma (MM) (Figure 3, Table 1). EBV PCR of a biopsy sample was sent which turned out to be negative. Lymphocyte subset analysis was done in view of suspected immunodeficiency which showed normal CD4, CD8, and NK cells.

**Table 1:** Immunohistochemical findings in the present case.

IHC Parameter	Findings
CD38	Strongly Positive
CD138	Strongly Positive
EMA (Epithelial Membrane Antigen)	Positive
Cyclin D1	Positive
Light Chain Expression	Monotypic lambda light chain restriction
Kappa Light Chain	Negative
CD20	Negative
CD45 (Leukocyte Common Antigen)	Negative
CD56	Negative
CD5	Positive in background T-cells
Ki-67 Proliferation Index	High (>80%)
EBV PCR	Negative

A whole-body low-dose CT was done which showed no lytic lesions. Bone marrow biopsy revealed all hematopoietic components with 3% plasma cells, without any evidence of granuloma or lymphoma deposits. Serum protein electrophoresis revealed an M-spike of 0.50 g/dL. The immunoglobulin profile revealed an elevated IgA level (360 mg/dL; normal range: 70 - 400 mg/Dl) with normal IgG and IgM, and Immunofixation revealed abnormal free light chains (kappa: 28 mg/L, lambda: 124.79 mg/L) with a kappa/lambda ratio of 0.225. Beta-2 microglobulin was also found to be elevated (3760 ng/mL; normal range: 600 - 2400 ng/mL).

Following surgical decompression and tumor excision, plasmablastic lymphoma was diagnosed as the absence of lytic bone lesions, low M-spike, and only 3% plasma cells in marrow pointed against MM or its plasmablastic transformation. The patient was initiated on the EPOCH chemotherapy regimen (etoposide, prednisone, vincristine, cyclophosphamide, doxorubicin) approximately two weeks postoperatively to allow for recovery. EPOCH was selected due to its established efficacy in treating aggressive lymphomas such as plasmablastic lymphoma. After two cycles (administered at 21-day intervals), the patient demonstrated notable symptomatic regression, including improved lower limb strength and reduced neurological deficits. 

In this case, the patient was HIV-negative, immunocompetent, and had localized paravertebral PBL with epidural extension but no leptomeningeal or systemic involvement. As there were no high-risk features for CNS relapse and the disease responded well to EPOCH chemotherapy, CNS prophylaxis was not administered. Instead, a strategy of close clinical and radiological monitoring was adopted to detect any early signs of CNS progression, given the focal nature and favorable early treatment response.

## Discussion

PBL and plasmablastic myeloma (PBM) represent rare and highly aggressive malignancies arising from plasmablasts, immature plasma cells with high proliferative capacity. PBM is a recognized morphological variant of MM, characterized by the proliferation of immature plasma cells (plasmablasts) exhibiting clear nuclei, prominent nucleoli, scant cytoplasm, and expression of plasma cell markers such as CD38 and CD138, along with light chain restriction [9]. Similarly, PBL shows an almost indistinguishable immunophenotypic profile, typically expressing CD38, CD138, MUM1, and EMA, with frequent absence of CD20 and CD45. This immunoprofile overlap complicates diagnostic differentiation based on histopathology alone. Due to overlapping clinical, morphological, and immunophenotypic features, distinguishing between these two entities remains a significant diagnostic challenge, particularly in immunocompetent individuals [10]. Vega et al. conducted a comparative immunohistochemical analysis of nine cases of PBL and seven cases of PBM, concluding that immunophenotyping alone is insufficient to reliably differentiate between the two (Table 2) [11]. The only notable discriminative marker identified was the association with Epstein-Barr Virus (EBV), which was more frequently observed in PBL, particularly in HIV-positive individuals.

**Table 2:** Diagnostic differences between plasmablastic lymphoma (PBL) and plasmablastic transformation of myeloma (PBM).

Feature	Plasmablastic Lymphoma (PBL)	Plasmablastic Transformation of Myeloma (PBM)
Clinical Setting	Common in immunosuppressed (HIV+, transplant)	Known or occult plasma cell dyscrasia
Typical Site	Extraosseous (oral cavity, GI, soft tissue, CNS)	Osseous lesions (spine, pelvis, skull)
Serum M-protein (SPEP)	Usually absent or low (<1 g/dL)	Typically present (M-spike)
Bone Marrow Plasma Cells	Absent or <10%	>10% clonal plasma cells
Lytic Bone Lesions	Rare	Common
CD138	Positive	Positive
CD38	Positive	Positive
CD45 (LCA)	Negative or weak	Usually positive
CD20	Negative or weak	Usually negative
CD79a	Variable (often weak or negative)	Positive
CD56	Often negative	Often positive
MUM1/IRF4	Positive	Positive
EBER (EBV-ISH)	Often positive (especially in HIV-positive cases)	Typically negative
Ki-67 Proliferation Index	High (>80%)	Variable (often lower)

In the current case, the patient presented with a subacute, insidious-onset spastic paraparesis secondary to thoracic spinal cord compression. MRI revealed a lobulated, enhancing soft tissue lesion in the paravertebral space from T1 to T6, with epidural extension, and without significant bony destruction, a radiological pattern more consistent with lymphoma than tuberculosis or MM. Given the patient’s prior history of pulmonary tuberculosis and partial ATT, a presumptive diagnosis of Pott’s spine was initially considered. However, the lack of vertebral body destruction, absence of disc involvement, and mass-like lesion on imaging prompted reconsideration and led to surgical decompression and histopathological confirmation.

Histopathology demonstrated large atypical lymphoid cells with immunoblastic and plasmablastic features. IHC was notable for CD38+, CD138+, EMA+, Cyclin D1+, lambda light chain restriction, and absence of CD20 and CD45 expression. These findings, in conjunction with the clinical context, immunocompetent host, absence of widespread marrow involvement, low M-protein level (M-spike 0.50 g/dL), and lack of lytic lesions, favored a diagnosis of HIV-negative PBL over plasmablastic transformation of myeloma (Table 2).

Notably, EBV PCR on biopsy tissue was negative. While EBV is frequently associated with PBL, especially in HIV-positive patients, EBV-negative PBL has been described in immunocompetent individuals, though it is less common and often portends a more aggressive course [12]. A recent comprehensive review of 402 HIV-negative cases found no significant survival benefit with intensive chemotherapy over CHOP-like regimens, and EBV-positivity remained the only independent prognostic factor affecting overall survival, underscoring the therapeutic challenge in this subgroup [6]. 

**Figure 4:**
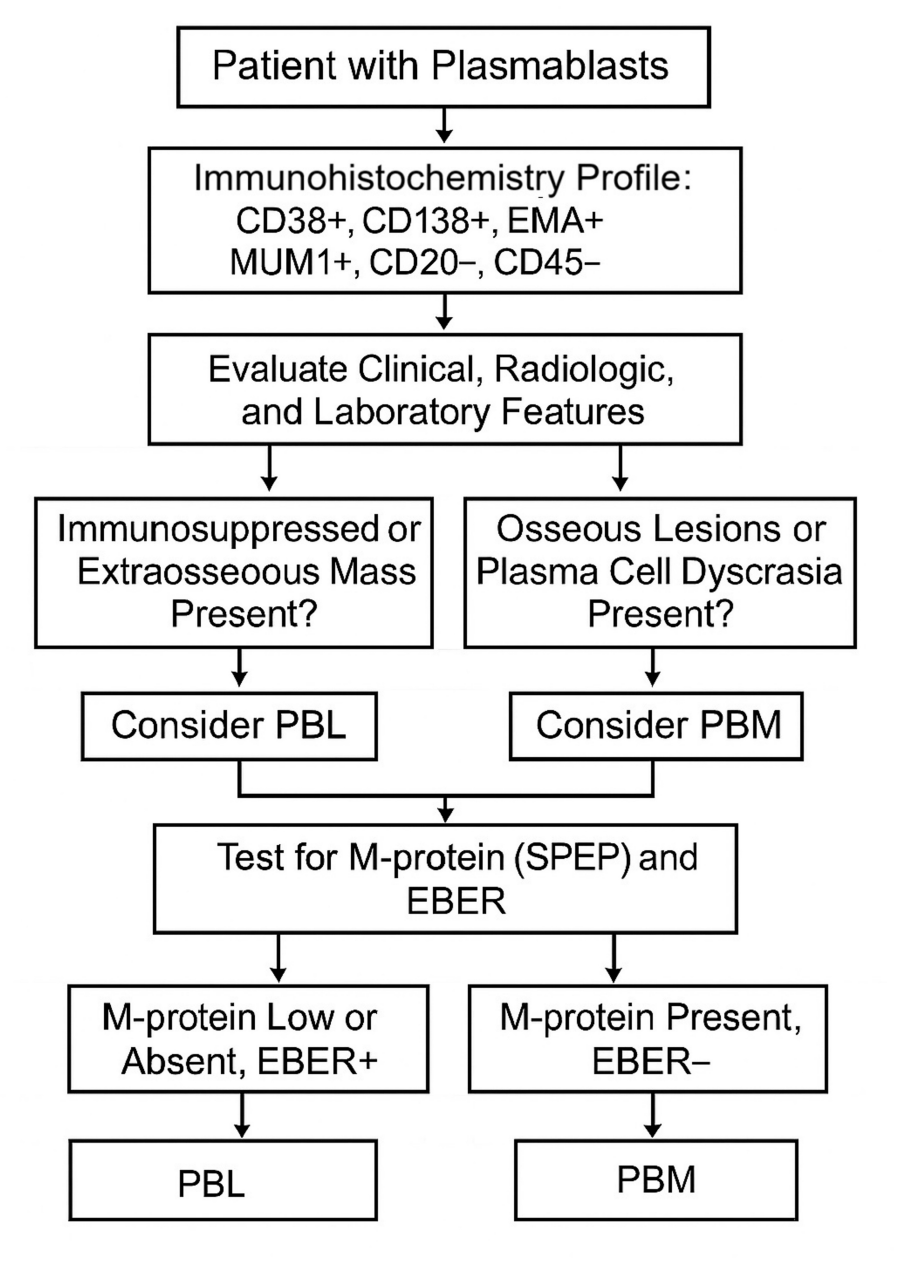
Algorithm for workup of patients with plasmablasts. PBL: Plasmablastic lymphoma; PBM: plasmablastic myeloma

Differentiating PBL from PBM or plasmablastic transformation of plasmacytoma is crucial due to divergent therapeutic approaches (Figure 4). PBL, due to its aggressive behavior and poor response to conventional CHOP-like regimens, typically requires more intensive chemotherapy protocols such as DA-EPOCH (dose-adjusted etoposide, prednisone, vincristine, cyclophosphamide, and doxorubicin) [3]. A meta-analysis of 12 studies involving 410 PBL patients showed that novel agents, especially bortezomib-combined with chemotherapy, significantly improve objective response and progression-free survival in newly diagnosed PBL, with no increase in adverse events [13]. Stem cell transplantation, radiotherapy, and emerging immunotherapies (e.g., CAR-T, PD-1 inhibitors) show promising results. 

In contrast, PBM or plasmablastic transformation of MM is generally managed using anti-myeloma regimens that incorporate proteasome inhibitors such as bortezomib and immunomodulatory drugs like lenalidomide, often followed by autologous stem cell transplantation (ASCT) in eligible patients. This treatment paradigm is extrapolated from the management of aggressive myeloma subtypes such as plasma cell leukemia (PCL), which shares overlapping features with PBM. According to the International Myeloma Working Group consensus, PCL requires prompt initiation of high-intensity therapy with bortezomib-based combinations followed by high-dose therapy and ASCT, or even allogeneic stem cell transplantation in selected younger patients [14]. 

In the present case, the patient underwent T2-T6 laminectomy and tumor excision to relieve spinal cord compression. Postoperative histological confirmation of PBL prompted initiation of the DA-EPOCH regimen. The patient tolerated chemotherapy well, and early symptomatic improvement in neurological function, particularly in the lower limb, was noted following two cycles.

Given the aggressive natural history of PBL, especially in HIV-negative patients, long-term surveillance is imperative. The patient has been enrolled in a structured follow-up program, including clinical assessments, laboratory monitoring (CBC, serum protein electrophoresis, immunoglobulin levels, free light chains), and imaging (MRI and/or PET-CT). Surveillance intervals have been planned every three months for the first year, then every six months for the next two years, and annually thereafter to detect early relapse or therapy-related complications.

## Conclusions

This case illustrates the diagnostic complexity of plasmablastic neoplasms, particularly in immunocompetent individuals with confounding clinical factors such as prior tuberculosis. It underscores the need for a broad differential diagnosis when evaluating spinal cord compression syndromes and highlights the value of early imaging and timely tissue diagnosis. Accurate classification of plasmablastic malignancies is critical, as it guides treatment strategies and influences prognosis. Timely surgical decompression combined with appropriate systemic chemotherapy can lead to meaningful neurological and clinical recovery, even in aggressive entities like HIV-negative PBL. It emphasizes the importance of considering PBL as a differential diagnosis, irrespective of immune status, to facilitate timely intervention and optimize patient outcomes.
